# A defined synthetic substrate for serum-free culture of human stem cell derived cardiomyocytes with improved functional maturity identified using combinatorial materials microarrays

**DOI:** 10.1016/j.biomaterials.2015.05.019

**Published:** 2015-05-15

**Authors:** Asha K. Patel, Adam D. Celiz, Divya Rajamohan, Daniel G. Anderson, Robert Langer, Martyn C. Davies, Morgan R. Alexander, Chris Denning

**Affiliations:** a Wolfson Centre for Stem Cells, Tissue Engineering and Modeling, University of Nottingham, Nottingham, NG7 2RD, UK; b David H. Koch Institute for Integrative Cancer Research, Massachusetts Institute of Technology, 500 Main Street, Cambridge, MA 02139, USA; c Laboratory of Biophysics and Surface Analysis, School of Pharmacy, University of Nottingham, Nottingham, NG7 2RD, UK; d Wyss Institute for Biologically Inspired Engineering at Harvard University, Boston, MA 02115, USA; e Department of Chemical Engineering, Massachusetts Institute of Technology, 500 Main Street, Cambridge, MA 02139, USA; f Institute for Medical Engineering and Science, Massachusetts Institute of Technology, 500 Main Street, Cambridge, MA 02139, USA; g Harvard-MIT Division of Health Science and Technology, Massachusetts Institute of Technology, 500 Main Street, Cambridge, MA 02139, USA

**Keywords:** Stem cell, Cardiomyocyte, Cell adhesion, Cell spreading, Electrophysiology, Surface analysis

## Abstract

Cardiomyocytes from human stem cells have applications in regenerative medicine and can provide models for heart disease and toxicity screening. Soluble components of the culture system such as growth factors within serum and insoluble components such as the substrate on which cells adhere to are important variables controlling the biological activity of cells. Using a combinatorial materials approach we develop a synthetic, chemically defined cellular niche for the support of functional cardiomyocytes derived from human embryonic stem cells (hESC-CMs) in a serum-free fully defined culture system. Almost 700 polymers were synthesized and evaluated for their utility as growth substrates. From this group, 20 polymers were identified that supported cardiomyocyte adhesion and spreading. The most promising 3 polymers were scaled up for extended culture of hESC-CMs for 15 days and were characterized using patch clamp electrophysiology and myofibril analysis to find that functional and structural phenotype was maintained on these synthetic substrates without the need for coating with extracellular matrix protein. In addition, we found that hESC-CMs cultured on a co-polymer of isobornyl methacrylate and *tert*-butylamino-ethyl methacrylate exhibited significantly longer sarcomeres relative to gelatin control. The potential utility of increased structural integrity was demonstrated in an *in vitro* toxicity assay that found an increase in detection sensitivity of myofibril disruption by the anti-cancer drug doxorubicin at a concentration of 0.05 µM in cardiomyocytes cultured on the co-polymer compared to 0.5 µM on gelatin. The chemical moieties identified in this large-scale screen provide chemically defined conditions for the culture and manipulation of hESC-CMs, as well as a framework for the rational design of superior biomaterials.

## 1. Introduction

The differentiation of hESCs to cardiomyocytes was first reported in 2000 [1] and has since undergone improvements in culture conditions to include staged addition of growth factors to increase differentiation efficiency [2], replacement of these factors with small molecules to reduce cost [3] and defined medium components to improve reproducibility [4]. However, progress in the development of defined and reproducible synthetic substrates has been limited by a lack of understanding of the cell–surface interactions that control cell phenotype. Pre-adsorption of extracellular matrix (ECM) proteins such as gelatin, laminin and fibronectin offer varying degrees of support for cardiomyocyte adhesion [5]. However, common synthetic substrates, such as tissue culture polystyrene, even when coated with ECM proteins, have been shown to cause catastrophic loss of sarcomeric integrity [6]. Biological substrates can be expensive, have a limited shelf life and are subject to batch variation. Such matrices are often assumed to be inert and their effect on cell behavior is over-looked [7]. In addition, undefined fetal bovine serum (FBS) commonly added to culture medium has been shown to alter phenotype thereby masking drug and disease effects [8]. Synthetic culture substrates, together with defined serum-free media components, could circumvent these concerns [9]. A wide chemical survey of the underlying substrate supporting the cells has not previously been investigated for their effect on hESC-CM adhesion and function.

High throughput screening (HTS) strategies for biomaterials development have proved successful in identifying substrates capable of supporting clinically relevant cell types including hESCs [10–13], pancreatic islet cells [14] and hepatocytes [15]. The current study has employed a parallel screening approach to investigate the influence of a range of (meth)acrylate and (meth)acrylamide polymers on hESC-CM adhesion and functionality. Members of this class of polymers were selected due to the large chemical diversity available commercially and because they are amenable to *in situ* free-radical polymerization. Over 1700 substrates can be presented in a single polymer microarray by depositing nano-liter volumes of monomer into discrete 300 µm islands by piezo or contact printing and polymerizing on-slide [16]. Coupled with high throughput surface characterization [17], high content imaging systems and automated image analysis [18], we present a powerful strategy to rapidly identify materials that support functional hESC-CMs in fully defined conditions and demonstrate potential applications for such a system in drug toxicity screening.

## 2. Materials and methods

### 2.1. Cell culture

#### EB differentiation

Cardiac differentiation was adapted from previously published protocols [19,20]. Briefly, embryoid body (EB) formation of the HUES7 [21] cell line was initiated in untreated polystyrene 96 V-well plates (NUNC, 249662) by seeding each well with 4000 cells in 100 µL of RPMI 1640 medium (Invitrogen) supplemented with 1× insulin transferrin selenium (Invitrogen), 1× chemically defined lipid (Invitrogen), 400 µM 1-thioglycerol (Sigma) (denoted RILT medium) plus 0.4% Poly(vinyl alcohol) (Sigma) and growth factors 20 ng/mL BMP-4 (R&D) and 6 ng/mL basic FGF (Peprotech) to direct differentiation to cardiomyocytes. Plates were incubated for 48 h at 37 °C, 5% CO_2_ and medium changed to RPMI 1640 supplemented with 20% FBS and incubated for a further 48 h. At day 4 of differentiation, EBs were transferred to a tissue culture polystyrene 96U-well plate (NUNC, 168136) in 150 ´L of RILT medium which was changed every 3 days. EBs began to spontaneously beat from day 8.

#### Monolayer differentiation

A previously published protocol [4] was followed. Briefly, HUES7 cells were seeded at a density of 1.2 × 10^4^ cells per cm^2^ in a tissue culture polystyrene T flask coated with Matrigel (BD Biosciences). Differentiation was initiated on day 4 using 6 µM of CHIR99021 (Tocris) in chemically defined medium (CDM) which comprises of RPMI 1640, 213 µg/mL of l-ascorbic acid-2-phosphate (Sigma–Aldrich) and 500 µg/mL of human recombinant albumin (Sigma–Aldrich). After 48 h, medium was changed to CDM containing 2 mM Wnt-C59 (Tocris). After a further 48 h, medium was changed to CDM and maintained in this medium for 2 days and then switched to RILT medium for maintenance. Spontaneous beating was observed between day 7 and 9 from initiation of differentiation.

### 2.2. Cardiomyocyte cluster disaggregation

Beating clusters of cells within EBs were dissected at day 15 of differentiation, washed in PBS and transferred to a mixture of 0.05% trypsin-EDTA and AccuMax (Innovative CellTech) in a 3:1 ratio and incubated for 8 min (with vortexing at 4 min intervals). Dissociation was confirmed with gentle pipetting. Partially dissociated clusters were transferred to fresh enzyme mix to repeat the incubation and vortex process. Meanwhile, the remaining enzyme-cell suspension was quenched with an equal volume of RPMI supplemented with 20% FBS and centrifuged for 3 min at 300G. The supernatant was gently aspirated and the cell pellet re-suspended in a small volume of RILT medium until all clusters were disaggregated and pooled together. Monolayer cultures were disaggregated using the same enzyme mixture with exposure reduced to 3 min in total followed by quenching and centrifugation steps as described above.

### 2.3. Polymer microarray synthesis

Polymer microarrays were fabricated as described previously [22]. Briefly, monomer solutions (Sigma Aldrich, Scientific Polymers and Polyscience) were spotted, using a XYZ3200 dispensing station (Biodot) and metal pins (946MP3B, Arrayit), onto epoxyglass slides (Genetix) dip-coated with pHEMA (4% w/v, Sigma) in ethanol (95% v/v in water). The printing conditions were O_2_ < 2000 ppm, 25 °C, and 35% humidity. Homopolymer solutions were composed of monomer (50% v/v) in dimethylformamide with photo-initiator 2,2-dimethoxy-2-phenyl acetophenone (1% w/v). Six replicates of 116 homopolymers were printed on each slide of a first generation array. The monomer portion of co-polymer solutions consisted of major monomer and minor monomer in a 30/70% v/v ratio. Three replicates of 576 co-polymers were printed in second generation arrays. Co-polymers were scaled up by piezo printing using a SciflexarrayerS11 (Scienion) onto 35 mm dishes (NUNC, 150318) that had been oxygen plasma etched at 30 W for 10 min (BioRad, PT7100) and coated with 4% pHEMA solution. Prior to seeding with cells, all substrates were UV sterilized, washed with phosphate buffered saline (PBS, Invitrogen) and incubated for 1 h with either RILT medium alone or supplemented with 20% Fetal Bovine Serum (FBS) (Invitrogen). The seeding density of cardiomyocytes was optimized at 80,000 cells per microarray to avoid high densities leading to very high cell counts which would lead to inaccurate automated image analysis (Fig. S1).

### 2.4. Whole cell patch clamp electrophysiology

Recordings were performed in current clamp mode using an ECP-10 HEKA amplifier. Cells were maintained in Normal Tyrodes buffer (140 mM NaCl, 10 mM glucose, 10 mM HEPES, 4 mM KCl, 1 mM MgCl_2_, 1.8 mM CaCl_2_, pH 7.45/NaOH) and at near-physiological temperatures (37 ± 2 °C). Patch pipettes were pulled on a Sutter P-97 programmable micropipette puller and had resistances of between 2 and 5 MΩ when filled with the internal solution (145 mM KCl, 5 mM NaCl, 2 mM CaCl_2_, 2 mM MgCl_2_, 4 mM EGTA, 10 mM HEPES, pH 7.3/KOH). Pulse (HEKA) and Clampfit v9.0 (Molecular Devices) software were used for data acquisition and analysis respectively. To determine the sub-type of the cardiomyocyte, APD90/50 ratios were calculated and designated as ventricular <1.4, nodal 1.4–1.7 and atrial >1.7. Ventricular subtypes were selected for further action potential profiling.

### 2.5. Immunostaining

Cells were fixed in 4% paraformaldehyde (Sigma) and permeabilized with 0.1% Triton-X 100 (Sigma). Non-specific binding was blocked with 4% goat serum (Dako) in PBS for 1 h. Samples were immunostained with primary antibody against human cardiac sarcomeric α actinin raised in mouse (1:800; Sigma). After 24 h and a 0.1% Tween20 (Sigma) wash, samples were exposed to Cy3-conjugated goat anti-mouse secondary antibody IgG + IgM (1:250; Jackson Immuno Research) and 4′,6-diamidino-2-phenylindole (DAPI) (1:1000; Sigma). Samples were mounted in VectorShield mounting medium (Vector Labs, Peterborough, UK) and imaged using an automated fluorescence microscope (IMSTAR).

### 2.6. Doxorubicin assay

hESC-CMs differentiated using the monolayer method were seeded onto the selected co-polymers or 0.1% gelatin coated dishes in RILT medium and incubated for 15 days with medium changes every 3 days. Doxorubicin (Cell Signaling) was diluted in dimethyl sulfoxide (DMSO, Hybri-Max, Sigma) to a stock concentration of 5 µM. This was diluted further in RILT medium to 0.05 µM, 0.5 µM and 5 µM concentrations. The final concentration of DMSO in each dilution was kept constant. Cells were treated with the doxorubicin spiked RILT medium at day 15 of culture and cells were incubated for 24 h before fixing for immunostaining. Disrupted myofibrils were considered to be sarcomere lengths of less than 1.4 µm [23] or punctate staining where no myofibril banding could be observed.

### 2.7. Image analysis

Automated image analysis of cardiomyocyte density, cell area and shape was achieved by building a custom protocol using Cell-Profiler open source software [18]. CellProfiler pipelines can be found at www.CellProfiler.org. The lengths of sarcomeres within myofibrils were measured using the line profile tool in Image J downloaded from http://imagej.nih.gov/ij/. Cells within images were randomly selected by ‘object number’ using x, y co-ordinates generated by CellProfiler software during individual cell analysis (Fig. S2.).

### 2.8. Time-of-flight secondary-ion mass spectrometry

ToF-SIMS analysis was carried out using a ToF-SIMS IV instrument (ION-TOF GmbH, Münster, Germany) using a $Bi_{3}^{+}$ primary ion source operated at 25 kV. A 1 pA pulsed primary ion beam was rastered and secondary ions were collected from a 10 × 10 mm area at a resolution of 100 pixels per mm, with 8 ion pulses per pixel. An ion dose of 2.45 × 10^11^ ions per cm^2^ was applied to each sample area ensuring static conditions. To compensate for a surface build up of positive primary ions, low energy electrons (20 eV) were delivered via a flood gun. Data analysis was carried out using Surfacelab6 software.

### 2.9. Partial least squares (PLS) multivariate linear regression

To correlate surface analytical data with cell response PLS was carried out using the Eigenvector PLS toolbox 5.2.2 for Matlab using the SIMPLS algorithm [24]. A peak list was generated for the homopolymer array consisting of 1397 ions. Mean-centered data preprocessing was applied and a leave-one-out cross validation was carried out to obtain errors for latent variables. The model was generated using 80% of the data (training set) and validated by predicting values for the remaining 20% of the data [25]. These models provide a means of study for systems where there is limited a priori knowledge and also removes subjective manual analysis of variables to create statistically valid models [26].

## 3. Results

### 3.1. Commercially available (meth)acrylate and (meth)acrylamide homopolymers are unable to support hESC-CM adhesion in serum-free conditions

**Fig. 1:**
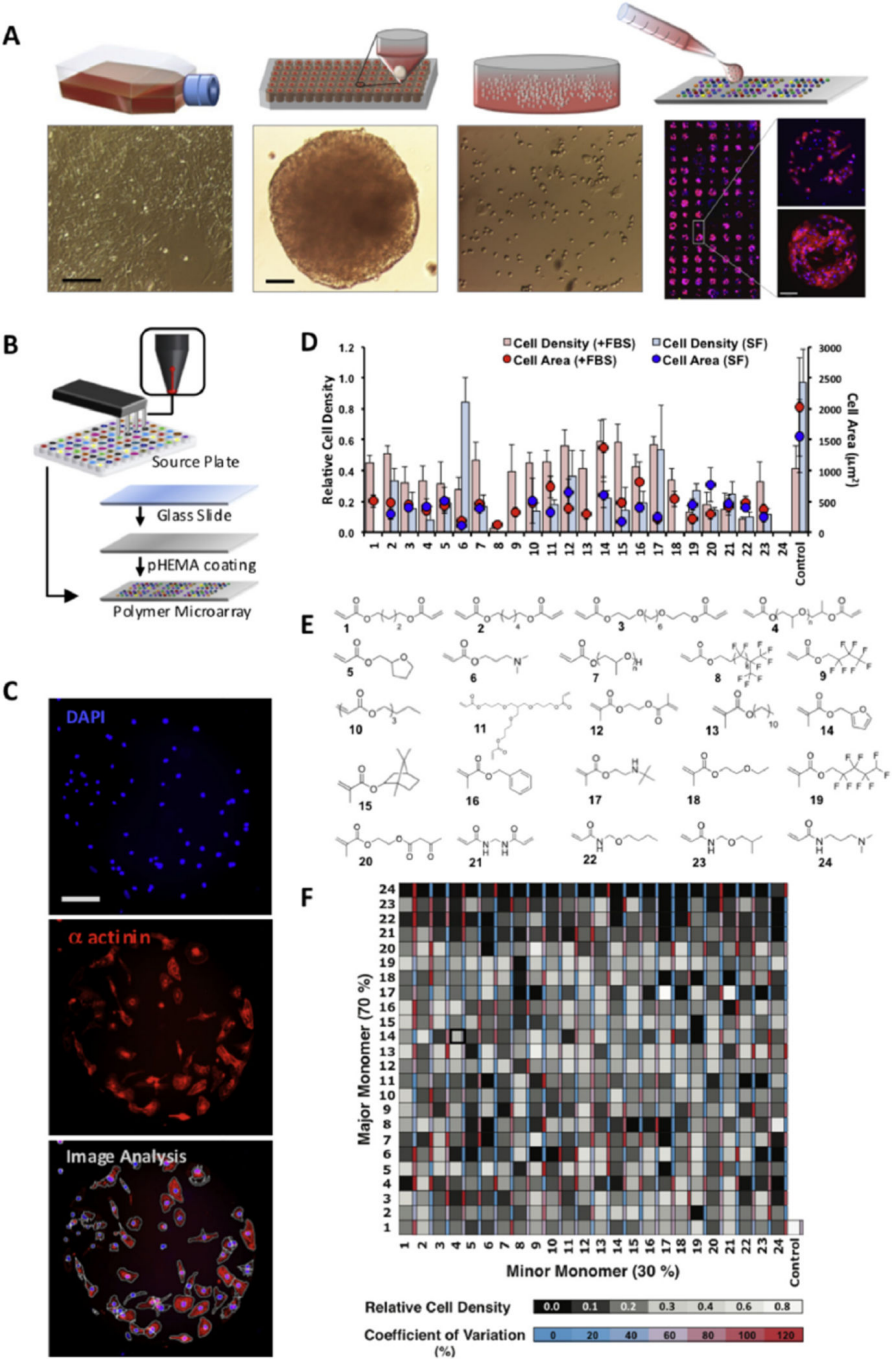
Polymer microarray fabrication, hESC-CM seeding and analysis. (A) hESC cardiomyocytes are derived via embryoid bodies and disaggregated to seed microarrays. (B) Contact printing was used to fabricate polymer microarrays on a glass slide coated with pHEMA to prevent background cell adhesion. (C) DAPI and cardiac sarcomeric α actinin images of cardiomyocyte adhesion were analyzed by CellProfiler^®^ to generate cell density and morphological data (D) Cell adhesion (bars) and size (circles) on 24 selected polymers from an initial 116 polymer screen in serum-free (SF, blue) and FBS pre-treated conditions (red). (n = 3, ±SEM). (E) Monomer identities. (F) These were mixed pair wise in 70/30 (v/v) mixtures to generate a 576 co-polymer microarray; heat map of cell adhesion is shown (n = 3). Scale bars = 100 µm. (For interpretation of the references to color in this figure legend, the reader is referred to the web version of this article.)

To investigate whether surface chemistry can influence cardiomyocyte adhesion and spreading, a library of 96 (meth)acrylate and 20 (meth)acrylamide monomers with various side chain chemistries (Table S1) were arrayed onto glass slides coated with poly(2-hydroxyethyl methacrylate) (pHEMA) to anchor spots and reduce background cell adhesion (Fig. 1). Beating clusters of hESC-CMs were dispersed and seeded onto arrays (Fig.1A) that had either been pre-conditioned with defined serum-free medium or medium supplemented with 20% fetal bovine serum (FBS) for 1 h. Cultures were maintained for 7 days on microarrays in serum-free medium before processing for immunostaining against the cardiac specific structural protein, α actinin, that resides in the Z bands of the myofibril and its staining pattern provides information on cell structure, size and maturity (Fig. 1C). Seeding densities were optimized to allow accurate automated image analysis using CellProfiler. Very high densities were found to lead to inaccurate counts and low densities failed to reveal differences in levels of cell adhesion on various polymers (Fig. S1). It was found that on arrays pre-conditioned with FBS, 48 of the 116 homopolymers supported hESC-CM adhesion (Fig. S3A). However, in the absence of serum, only 7 supported adhesion to a level comparable with the gelatin control (Fig. S3B), underlining the dependency on FBS for adhesion. The highest number of attached cells in serum-free conditions was on two structurally related amine-containing polyacrylates; *tert*-butylamino-ethyl methacrylate (*monomer 17*) and dimethylaminopropyl acrylate (*monomer 6*) (Fig. 1D). However, average cell size was low (274 µm^2^ and 110 µm^2^ respectively) compared to cardiomyocytes on gelatin control (1560 µm^2^). Cardiomyocytes with an average cell size greater than 500 µm^2^ is desired as they represent more mature, later stage cardiomyocytes that are more likely to contain well-organized structural protein for functional cell contraction [27]. Poly(2-(methacryloyloxy)ethyl acetoacetate) (*monomer 20*) supported the largest average cell size (769 µm^2^) in serum-free conditions but had low cell attachment numbers. On serum pre-conditioned arrays, where we assume the surface is dominated by the adsorbed proteins [28], there was a diverse range of adhesion and spreading which is consistent with the observation that surface chemistry can alter protein adsorption and/or conformation that subsequently influences cell adhesion [29,30]. For example, when cultured with serum proteins, poly(furfuryl methacrylate) (*monomer 14*) is capable of supporting greater cell adhesion compared to poly(2-(methacryloyloxy)ethyl acetoacetate) (*monomer 20*) (Fig. 1D). However, in serum-free conditions, both polymers supported similarly low densities of cardiomyocytes. This initial screen identified polymers that demonstrated either high cell attachment or larger cell size but not both, consequently we sought to investigate if co-polymerization could capture both desired traits.

### 3.2. Combinatorial development of co-polymers enables improved cardiomyocyte adhesion and cell spreading in serum-free conditions

To investigate if attachment and cell spreading in serum-free conditions could be improved, we selected 24 polymers from both serum-free and serum pre-conditioned arrays. Homopolymers with highest attachment (>0.4 relative to highest number of cells/mm^2^) or cell size (>500 µm^2^) and an inter-replication variability co-efficient of <60% were selected for the second generation array. In addition, we selected 2 polymers that did not support cell adhesion in either condition (monomers *8* and *24*) and 4 polymers that supported adhesion on serum treated arrays but not in serum-free conditions (monomers *1, 9, 13* and *18*) (Fig. 1D). These 24 monomers were mixed pair-wise in 70/30 (% v/v) ratios to produce a combinatorial array of 576 co-polymers and as for the first generation array, we seeded with EB derived hESC-CMs and cultured for 7 days prior to assessment using α-actinin staining (Fig. 1C). A total of 20 of these unique 576 co-polymers supported high levels of relative attachment (>0.4) (Fig. 1F) and average cell size (>500 µm^2^) (Fig. S4). Poly (dimethylamino propyl acrylamide) (*monomer 24*) was identified to have a dominant negative influence on cell adhesion in every co-polymer mixture where it was the major monomer at 70% v/v (Fig. 1F). However, other trends were less obvious and required further systematic analysis.

### 3.3. Surface analysis and statistical modeling identifies chemical moieties that influence cardiomyocyte adhesion

**Fig. 2:**
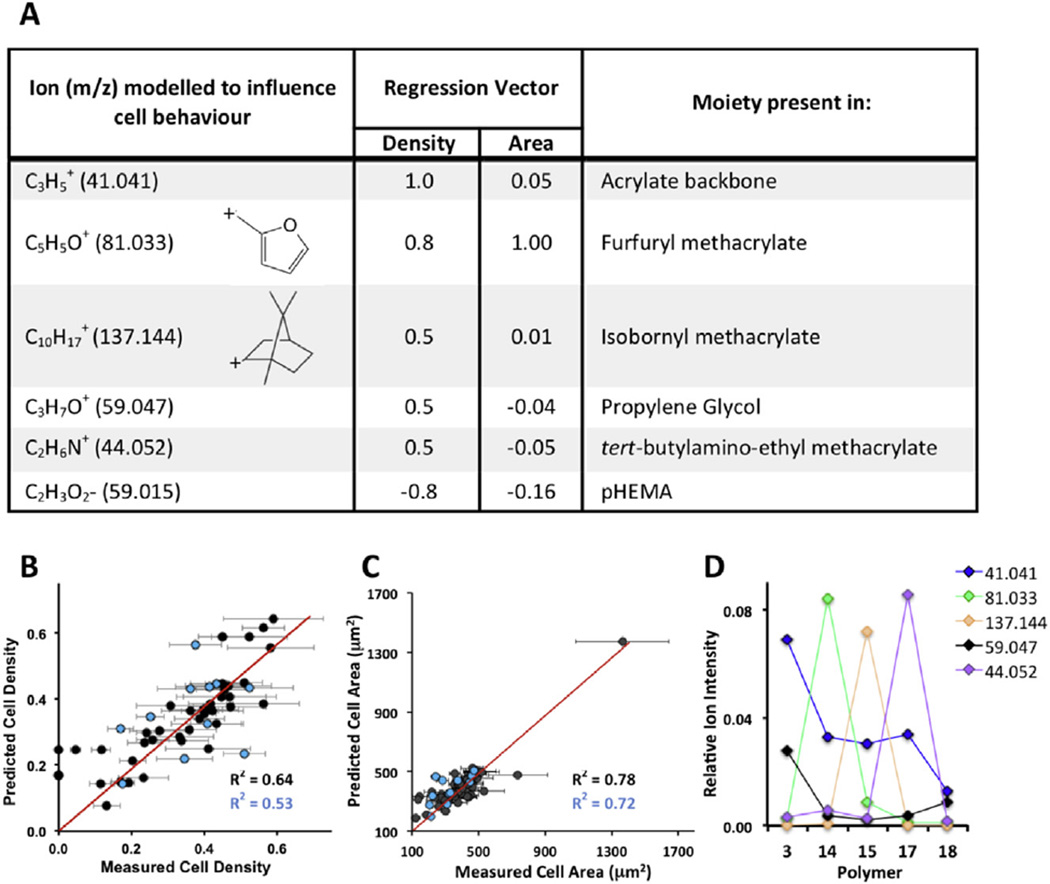
Partial least squares multivariate linear regression. (A) The table lists ions identified by the model to be important in influencing cell density or cell size (area). A positive regression vector (RV) describes the ion having an additive effect on density or cell area and a negative RV describes the ion as being detrimental on cell adhesion or area. For example while ion C_2_H_6_N^+^ has been modeled to improve cell density, it has a negative impact on cell area. (B) The model for predicting cell density has an R^2^ value of 0.64 (training data set, black). The model was validated using data that had not been introduced during training of the model (test set, blue). The R^2^ value for the test set is 0.53. (C) For cell area the R^2^ value is 0.78 (training set) and 0.72 (test set). (D) Surface ion intensity found by ToF-SIMS of moieties identified by PLS has been plotted for selected polymers where they had the highest relative intensities. (For interpretation of the references to color in this figure legend, the reader is referred to the web version of this article.)

Since a subset of polymers could improve hESC-CM adhesion and morphology, we aimed to determine which substrate properties were important in controlling cell behavior. Surface elemental composition data determined by X-ray photoelectron spectroscopy (XPS) and hydrophilicity measured by water contact angle (WCA), did not correlate to differences observed in cardiomyocyte adhesion and spreading (Fig. S6). In agreement with previous studies [28,31], WCA measurements revealed that the highest cell adhesion and spreading (cell size) was seen on polymers that had a WCA between 60°–80°, although lower cell adhesion and size was also observed within this range indicating WCA alone could not determine cell response to the substrate. To probe surface chemistry in greater detail, time-of-flight secondary-ion mass spectrometry (ToF-SIMS) was carried out to identify the important chemical moieties in the uppermost 2 nm of the surface. Surface characterization is essential to confirm the identity of surface chemistry available for cellular interaction, which may be different to the bulk chemistry [12]. To establish if there was any correlation between polymer surface chemistry and cell behavior, multivariate linear regression was employed as described in detail elsewhere [32–34]. The analysis found that there was a correlation between surface chemistry and cardiomyocyte adhesion (R^2^ = 0.64) and cell size (R^2^ = 0.78) (Fig. 2). Over 1300 positive and negative secondary ions detected from each substrate on the microarray by ToF-SIMS were assigned a regression vector (RV) to describe their effect on cell behavior. Large positive vectors indicated a positive effect of these secondary ions on cell density or area, whereas secondary ions assigned a negative value are associated with a detrimental effect on the cell response. The secondary ion C_2_H_6_N^+^ was assigned vectors of 0.5 for density and −0.05 for area (Fig. 2). This ion was most intense in the polymer *17* and mirrors experimental data where adhesion to the polymer was relatively high but cell area was amongst the lowest (Fig. 1C). The cyclic ions C_5_H_5_O^+^ and $C_{10}H_{17}^{+}$, most intense from polymers of *14* and *15* respectively (Fig. 2D), were identified as having positive effects on both cell adhesion/area with RV's of 0.8/1.00 and 0.5/0.01 respectively (Fig. 2A). The secondary ion $C_{2}H_{3}O_{2}^{-}$, which was most intense from pHEMA, was assigned negative RVs for both cell density and area (−0.8/−0.16). This indicates pHEMA from the underlying slide was contaminating the surface of some polymer islands. Detection of this contaminant highlights the importance of characterizing surface chemistry rather than assuming its identity from the monomer composition.

**Fig. 3:**
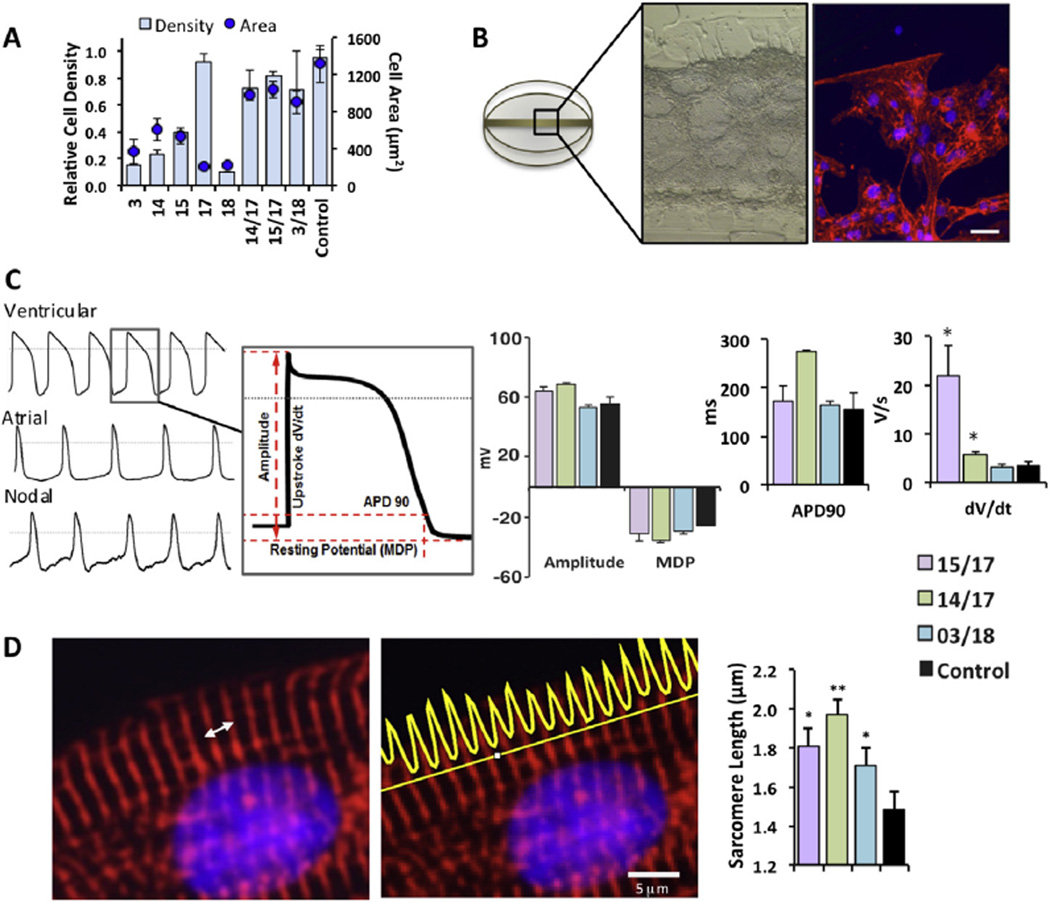
Structural and electrophysiological characterization of hESC-CMs after 15 days culture on co-polymers and control 0.1% gelatin (A) Cardiomyocyte adhesion and cell size on selected co-polymers compared to their constituent homopolymers (n = 4, ±SEM). (B) The three co-polymers were scaled up in 35 mm dishes, a brightfield image of hPSC-CM is shown with the corresponding immunostained image, α actinin (red) and DAPI (blue). (C) Ventricular, atrial and nodal-like action potentials (AP) were obtained using whole cell patch clamp electrophysiology. Characterization of ventricular AP parameters reveals that upstroke velocity is faster in cardiomyocytes cultured on polymers *15/17* and *14/17* relative to control. No statistical difference was observed in action potential duration at 90% repolarization (APD90), amplitude and maximal diastolic potential (MDP). T-Test, p < 0.05, 15/17 n = 5, 14/17 n = 4, 03/18 n = 3, control n = 3 cells, +SEM. (D) Structural analysis of the cardiomyocyte; the white arrow highlights one sarcomere unit. The length of each sarcomere was measured using Image J line profile tool (yellow intensity graph). Structural maturity was improved as indicated by longer sarcomere length in cardiomyocytes cultured on the synthetic polymers compared to control (3 measurements per cell, 4 cells measured per image, n = 3, +SD, T test, *p < 0.05, **p < 0.005). (For interpretation of the references to color in this figure legend, the reader is referred to the web version of this article.)

To validate the robustness of the model, 20% of substrate data that were not included when generating the model were then used as a test data set [25]. The model was able to predict cell adhesion (R^2^ = 0.53) and area (R^2^ = 0.72) based only on the surface chemical characterization of these substrates acquired using ToF-SIMS (Fig. 2B, C). The chemical moieties, C_2_H_6_N^+^, C_5_H_5_O^+^ and $C_{10}H_{17}^{+}$, which dominate the positive cell response on the array, may serve useful for tailoring substrate chemistry to manipulate cardiomyocyte adhesion and morphology and to gain greater understanding of cell–substrate interactions. It is also important to appreciate the subtle additive effects of ions that are assigned smaller RVs. For example, polymer *18* does not contain high intensities of the ions identified in Fig. 2A but when co-polymerized with monomer *3* contributes to an overall improvement of cardiomyocyte adhesion and spread compared to either homopolymer alone (Fig. 3A).

### 3.4. Electrophysiological function is maintained in hESC-CMs cultured on hit co-polymers in serum-free conditions

We examined hESC-CM structure and function in detail on 3 co-polymers; furfuryl methacrylate (70% v/v) with *tert*-butylaminoethyl methacrylate at 30% v/v (*14/17*), isobornyl methacrylate mixed with the same minor monomer (*15/17*) and hexanediol ethoxylate diacrylate polymerized with ethoxyethyl methacrylate (*3/18*). These were selected because they were amongst the top performing co-polymers where the performance of the co-polymer exceeded that of the constitutive homopolymers (Fig. 3A) and contained chemical moieties that were identified by multivariate analysis to be of importance (Fig. 2D). The co-polymers of *14/17, 15/17* and *3/18* supported high cardiomyocyte densities (0.4, 0.5 and 0.7 respectively) as well as larger cell size (977 µm^2^, 1033 µm^2^ and 899 µm^2^ respectively) (Fig. 3A). There is growing interest within the pharmaceutical industry to use hESC-CMs in drug safety evaluation to detect fatal drug-induced ventricular arrhythmias, such as *Torsade de Pointes* [35]. Since electrophysiological function is vital for this use we tested whether synthetic polymers could support beating cardiomyocytes. We scaled up co-polymers by piezo-printing a 600 µm wide line of polymer across the center of 35 mm dishes (Fig. 3B) that had been prepared by oxygen plasma etching followed by pHEMA coating. hESC-CMs were cultured on these substrates for 15 days and then subjected to whole cell patch clamp electrophysiology. Ventricular sub-types were analyzed to find that action potential duration at 90% repolarization (APD90), amplitude and maximal diastolic potential were comparable to hESC-CMs on gelatin controls, the upstroke velocity increased six-fold on co-polymer *15/17* (21.8 V/s, P < 0.05) and almost two-fold on *14/17* (5.8 V/s, P < 0.05) compared to hESC-CMs cultured on gelatin control (3.4 V/s) (Fig. 3C). Despite this modest improvement in velocity compared to values reported for primary human fetal cardiomyocytes (~8 V/s) [36], overall electrophysiological maturity remains low compared to adult human cardiomyocytes (~250 V/s) [36,37] similar to values reported for other stem cell derived cardiomyocytes, which also exhibit immature action potential profiles [38,39]. Adding maturity-promoting factors to the minimally complex medium we selected for this study could be a strategy to systematically test factors without confounding cues arising from chemically undefined substrates.

### 3.5. Synthetic substrates support cardiomyocytes with improved myofibril organization and evaluation of drug toxicity caused by myofibril disruption

**Fig. 4:**
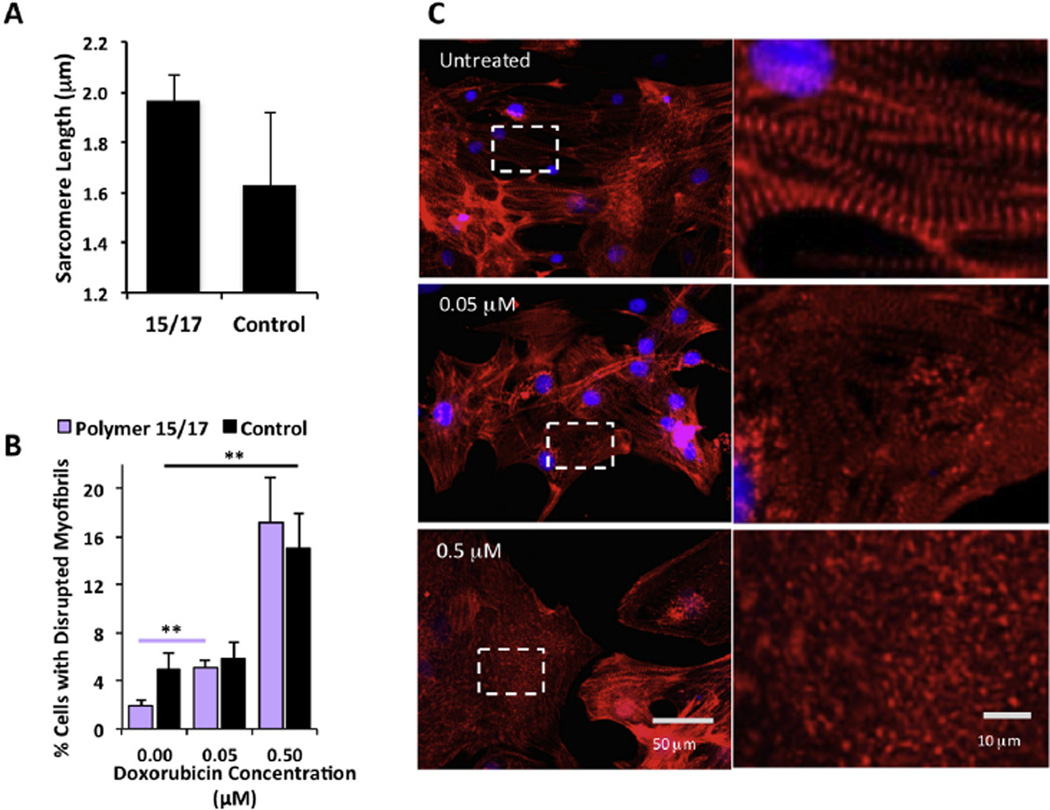
Structural Characterization of monolayer derived hESC-CMs after 15 days culture on co-polymer 15/17 compared to control 0.1% gelatin and detection of myofibril disruption by doxorubicin. (A) Structural organization of hESC-CMs was improved as indicated by longer sarcomere length on polymer 15/17 with lower deviation compared to 0.1% gelatin control (3 measurements per cell, 4 cells measured per image, n = 3, +SD. (B) Cardiomyocytes on the synthetic polymer 15/17 have a more organized sarcomeres making disruption in their myofibril organization significant at 0.05 µM compared to control cardiomyocytes on gelatin where disruption of myofibrils is not statistically detected at 0.05 µM (n = 3, 250 cells analyzed per condition). (C) α actinin (red) and DAPI (blue) immunostains reveal that untreated cardiomyocytes on polymer 15/17 contain organized myofibrils. At a dose of 0.05 µM doxorubicin ‘pre-myofibrils’ with sarcomere lengths of less than 1.4 µm can be observed and at a dose of 0.5 µM, more obvious punctate staining with absence of myofibril alignment is seen. (For interpretation of the references to color in this figure legend, the reader is referred to the web version of this article.)

Cardiomyocytes were examined for α-actinin staining patterns as a rapid gauge of structural integrity. The distance between the Z bands correspond to sarcomere length, the basic motor units that make up the myofibril. Longer sarcomeres of up to 2.2 µm indicate structural maturity of the myocyte [40] and correlates to contractile functionality as determined by the Frank Starling mechanism [41]. Relative to control hESC-CMs on gelatin (sarcomere length 1.50 µm), those on co-polymers *14/17, 15/17* and *3/18* had significantly longer lengths of 1.97 µm (p < 0.005), 1.80 µm (p < 0.05) and 1.70 µm (p < 0.05) respectively (Fig. 3D). To assess if hESC-CMs derived from an alternative method could also maintain structural integrity on the synthetic polymer, we used a monolayer protocol to derive hESC-CMs and seeded them on polymer *15/17*. hESC-CMs on control displayed high variability in sarcomere length (1.63 µm ± 0.29) compared to hESC-CMs on polymer *15/17* where myofibrils had a more consistent length of (1.97 µm ± 0.095) (Fig. 4A). We reasoned that the utility of improved sarcomeric organization would be demonstrated in detecting toxicity of drugs that affect cell structure. The anti-cancer drug doxorubicin can cause cardiotoxicity at therapeutic concentrations of 0.01–0.04 µM [42,43], one indication of this toxicity is myofibril disruptions [44]. However, *in vitro* assays, using rat, human and mouse cells can only detect structural changes at 0.5 µM and higher [45–47]. Disruption of sarcomeric organization was defined as a sarcomere length below 1.4 µm indicating ‘pre-myofibrils’ [23] or punctate α-actinin staining where no myofibril banding could be observed. In accordance with the literature, our study also found that disruption of sarcomeric structure of hESC-CMs cultured on gelatin could be observed at 0.5 µM (P < 0.005) but not at 0.05 µM (Fig. 4B, C). The higher reproducibility of myofibril alignment in hESC-CMs on polymer *15/17* meant that perturbation could be detected at the lower and more relevant therapeutic dose of 0.05 µM (P < 0.005). This represents up to a 10-fold improvement in detection sensitivity to doxorubicin of hESC-CMs cultured on synthetic polymers relative to those on gelatin or other *in vitro* systems available.

## 4. Discussion

The comparison of various substrates for supporting cardiomyocytes has previously been investigated by banding surfaces into broad groups of positively/negatively charged, acid/base or hydrophilic/hydrophobic chemistries [48,49]. In this study, using a combination of unbiased parallel screening and systematic statistical modeling, an unprecedented library of polymers were surveyed to identify specific chemical groups C_2_H_6_N^+^ (amine), C_5_H_5_O^+^ (furan ring) and $C_{10}H_{17}^{+}$ (isobornyl ring) that improve the adhesion density of human cardiomyocytes in serum-free conditions.

The amine functionality in poly(*tert*-butylamino ethyl methacrylate), is positively charged at physiological pH [50] and ionic interaction with negatively charged cell membrane proteins are thought to facilitate cell adhesion [51]. The mechanism of how the cyclic moieties help to maintain cardiomyocyte function *in vitro* is yet to be elucidated but provides the basis for tailored design of culture substrates that could not have been predicted from existing knowledge of cell–material interactions.

Substrates reported in the literature to support cardiomyocyte adhesion do so under conditions that include FBS in the culture medium, which aids adhesion by coating the substrate in proteins such as vitronectin [52]. Comparison of various substrates can be distorted by variations in the undefined components within FBS. In this study, the removal of serum from culture meant that confounding components were eliminated. Although this reduced the proportion of homopolymers that were able to support cell adhesion, combinatorial mixtures of polymers that were found to support either greater adhesion or larger cell area, increased the diversity of substrates able to support functional cardiomyocytes in serum-free conditions.

At 7 day (Fig. S5) and 15 day time points, a co-polymer of isobornyl methacrylate and *tert*-butylamino-ethyl methacrylate was identified to support cardiomyocytes with sarcomere lengths that were significantly longer with lower deviation than cardiomyocytes on control gelatin. The improvement in myofibril organization was demonstrated in hESC-CMs derived from embryoid body and monolayer differentiation methods. The utility of greater sarcomeric organization was exemplified by increased sensitivity of toxicity detection to the anti-cancer drug, doxorubicin, demonstrating the need for reproducible culture of cardiomyocytes in order for them to be used reliably for pharmacological assays. Importantly, our studies used human origin cardiomyocytes so that the findings are directly translatable and not subject to reported differences such as rat cardiomyocytes being able to adhere to a greater proportion of substrates compared to the more fastidious human cardiomyocyte [53]. Challenges remain in optimizing the overall culture system to obtain electrophysiological profiles comparable to those reported for adult human cardiomyocytes. Exploitation of polymer structure–function relationships identified in this study to maintain cardiomyocytes on chemically defined substrates, addition of soluble cues that promote maturation and the move into 3D systems will be explored to overcome this challenge.

## 5. Conclusions

The materials investigated in this study provide a defined, reproducible and economically viable alternative to biological matrices and their discovery could not have been predicted from existing knowledge of cell–material interactions.

The identification of chemically characterized cardio-supportive moieties provides the basis for the rational design of substrates to build into controlled culture systems where further improvement of cell maturation using soluble cues or 3D design (e.g. culture medium components and the physical form of the substrate) can be systematically investigated without the contribution of uncharacterized cues arising from biological matrices.

## Supplementary Material

Supplemental
